# Supplementary material for: Thermal transport of helium-3 in a strongly confining channel
Source: Nat Commun. 2020 Sep 24;11:4843. doi: 10.1038/s41467-020-18662-8 (PMC7515880; doi:10.1038/s41467-020-18662-8)
Supplement: Supplementary file 1 — Supplementary Information [file 41467_2020_18662_MOESM1_ESM.pdf]

# Supplementary Information

# Thermal transport of helium-3 in a strongly confining channel

Lotnyk *et al.*

# Supplementary Information: Thermal transport of helium-3 in a strongly confining channel

D. Lotnyk[1], A. Eyal[1,2], N. Zhelev[1], T.S. Abhilash[1,3], E.N. Smith[1], M. Terilli[1], J. Wilson[1,4], E. Mueller[1], D. Einzel[5], J. Saunders[6] and J.M. Parpia[1]

[1] *Department of Physics, Cornell University, Ithaca, NY, 14853, USA*

[2] *Physics Department, Technion, Haifa, Israel*

[3] *VTT Technical Research Centre of Finland Ltd, Espoo, Finland*

[4] *SUNY Geneseo, Geneseo, NY, 14454, USA*

[5] *Walther Meissner Institut, Garching, Germany*

[6] *Department of Physics, Royal Holloway University of London, Egham, TW20 0EX, Surrey, UK*

August 30, 2020

**Supplementary Note 1. Volume Flow and Heat Flow in a confined channel.**

We estimate the fountain pressure that results from the inflow of superfluid in response to heat applied to the fork in the isolated chamber via the London equation[1]:

$$\Delta P = \rho S \Delta T, \tag{1}$$

where $\Delta P$ [Pa] is the fountain pressure generated, $\rho$ [kgm$^{-3}$] is the mass density, $S$ [JK$^{-1}$kg$^{-1}$] the entropy density of the liquid and $\Delta T$ [K] is the temperature difference along the channel. For 22 bar at 1.15 mK (0.5 $T_c$), we estimate that for a 25 $\mu$K temperature difference, the fountain pressure would be of order 8.6 $\times$ $10^{-3}$ Pa using values for $S$ derived from the literature[2]. Corresponding values for the pressure difference generated in response to the 25 $\mu$K temperature difference at 0.62, 0 bar are listed in Supplementary Table 1 at $T/T_c$= 0.5. The pressure differences at low pressure are smaller by about a factor of 4, reflecting the entropy and temperature of the liquid at low pressure. Such small pressure differences would be difficult to measure using direct means, especially given the requirement of a small volume in the isolated chamber.

The size dependent effective viscosity, $\eta_{\mathrm{EFF}}$ is related to the bulk viscosity ($\eta_{\mathrm{bulk}}$) by a viscous mean free path, $\lambda_\eta$ dependent scaling factor, "$s$", so that $\eta_{\mathrm{EFF}} = s\eta_{\mathrm{bulk}}$. In the past[3], the factor $s$ had been calculated for a 135 $\mu$m tall channel but can be adapted to the 1.1 $\mu$m channel from knowledge of the Knudsen number, $K_n = \lambda_\eta/d$, where $d$ is the channel height. We read off $\lambda_\eta$ from Figure 8 for each of the 3 pressures at 0.5 $T/T_c$, and calculate the corresponding $K_{\mathrm{n}}$. The Knudsen number is then used to estimate the correction factor $s$. A simplified expression for the upper bound for the correction factor is given by $s = 1/(1 + 2K_{\mathrm{n}})$. For the Supplemen-

tary Table 1, we computed the correction factor based on the graphs in Reference [3]. For 0 bar we found the limiting (large Knudsen number behavior) to be $s = 0.37 \times K_{\mathrm{n}}^{-1.5}$ and for 22 bar $s = 0.09 \times K_{\mathrm{n}}^{-1.33}$, where we have included the effects of Andreev scattering from surfaces as described in the literature[3]. The values for $\eta_{\mathrm{bulk}}$ [Pa·s], and $K_{\mathrm{n}}$, and corresponding $s$ values are listed in Supplementary Table 1. We note that in Reference [[3]] it was found that at the lowest temperature and pressure, the experimental results showed more slip than accounted for by theory. This would imply that under strong confinement, the scaling factor $s$ may underestimate slip.

To compute the heat conducted, we first need an estimate of the volume rate of flow of normal fluid ($dV/dT$). This is established by geometrical properties, the fountain pressure $\Delta P$ (from Supplementary Equation 1) and $\eta_{\mathrm{EFF}}$.

$$dV/dt = \Delta P \cdot Z^{-1} \eta_{\mathrm{EFF}}^{-1}, \tag{2}$$

where the impedance for a rectangular channel, $Z$ = 12 $l/wd^3$ is defined by its length $l$ = 100 $\mu$m, width $w$ = 3 mm, and height $d$ = 1.1 $\mu$m, resulting in $Z$ = 3$\times$10$^{17}$ m$^{-3}$. The result for the volume flow is also listed in Supplementary Table 1.

The heat transfer rate that would result from the back flow of normal fluid can be written as:

$$\frac{dQ}{dt} = \rho T S(T) \frac{dV}{dt} = \frac{T \rho^2 S^2(T) \Delta T}{Z \eta_{\mathrm{EFF}}}. \tag{3}$$

We used Supplementary Equation 3 to calculate the resulting heat flow $dQ/dt$. It is evident from

| Pressure [bar] | $T/T_c$ | $\Delta P$/25$\mu$K [Pa] | $\eta_{bulk}$ [Pa·s] | $\lambda_\eta[cm]$ | $K_n$ | $s$ | $\eta_{EFF}$ [Pa·s] | $dV/dt$ [m$^3 s^{-1}$] |
|---|---|---|---|---|---|---|---|---|
| 0 | 0.5 | $1.8\times10^{-3}$ | 0.036 | $2.5\times10^{-2}$ | 230 | $1.0\times10^{-4}$ | $3.6\times10^{-6}$ | $1.7\times10^{-15}$ |
| 0.62 | 0.5 | $2.1\times10^{-3}$ | 0.030 | $2.0\times10^{-2}$ | 180 | $1.5\times10^{-4}$ | $4.5\times10^{-6}$ | $1.6\times10^{-15}$ |
| 22.0 | 0.5 | $8.6\times10^{-3}$ | 0.0021 | $1.25\times10^{-3}$ | 11 | $3.7\times10^{-3}$ | $7.8\times10^{-6}$ | $3.8\times10^{-15}$ |

Table 1: **Bulk, Effective Viscosity and Flow with 25 $\mu$K difference across channel** The pressure difference, Bulk viscosity, Viscous mean free path, Knudsen number, correction factor "$s$", and effective viscosity, together with volume flow resulting from a 25 $\mu$K temperature difference for the three different pressures studied, all calculated at 0.5$T_c$.

| Pressure [bar] | Density [kg·m$^{-3}$] | Flow [m$^3$·s$^{-1}$] | Temperature at 0.5 $T_c$ [K] | Entropy Density [J/K·Kg] | $dQ/dt$ [W] |
|---|---|---|---|---|---|
| 0 | 81.4 | $1.7\times10^{-15}$ | $4.64\times10^{-4}$ | 0.90 | $5.8\times10^{-17}$ |
| 0.62 | 83.0 | $1.6\times10^{-15}$ | $5.06\times10^{-4}$ | 0.99 | $6.7\times10^{-17}$ |
| 22.0 | 110 | $3.8\times10^{-15}$ | $1.15\times10^{-3}$ | 3.15 | $1.5\times10^{-15}$ |

Table 2: **Entropy and Heat Flow with 25 $\mu$K difference across channel** The density, volume flow (from Supplementary Table 1), temperature and Entropy density computed from Reference [2] to yield the expected heat flow from Supplementary Equation 3.

the results in Supplementary Table 2 that the thus calculated heat flow (accounting for slip) is much too small to initiate any observable equilibrium temperature increase in the HEC fork. The estimated values of the hydrodynamic thermal conductivity $\kappa_{\rm HYD}$ compared to the measured value of $\kappa(T_{\rm c})$ are of order $10^{-4}$ (or smaller) at all pressures and temperatures and therefore negligible. We show the calculated $\kappa_{\rm HYD}$ at 0 bar and at 22 bar using both the simplified correction factors for $s$ and the value of $s$ from Reference [3] in Supplementary Figure 1.

We can estimate when the thermal conductivity of the diffusive and hydrodynamic (counter-flow) terms might be comparable. In a large enough channel (mm size) we expect $\eta_{\rm EFF} \to \eta_{\rm bulk}$ ($s$=1). Comparing Supplementary Figure 1 to Figure 5(b) in the main text, the calculated thermal conductivity $\kappa_{\rm HYD} \propto Z^{-1}\eta_{\rm EFF}^{-1}$ is $\sim 10^{-4}$ smaller than the measured $\kappa_{\rm EFF}$ for a 1.1 $\mu$m channel. Since $\eta_{\rm EFF}$ is reduced by a factor of $10^{-4}$ in the Knudsen regime (Supplementary Table 1), scaling argues that $Z$ would have to decrease by $10^{-8}$ to make the two thermal conductivity contributions comparable. Since $Z \propto d^{-3}$, the height $D$ for comparable thermal contributions would be $\sim 10^3 d$ or $D =\sim$ 1 mm.

**Supplementary Note 2. Particle flow during and after pulse.**

During the pulse applied to a fork, heat is generated by the motion of the fork's tines as they interact with the $^3$He. That heat results in a temperature rise, $\Delta T$ in the chamber that the fork is situated within. For definiteness here, we assume this is the IC. For simplicity, we assume the temperature rise to be linear in time so $dT/dt$ is constant. The number of $^3$He particles in the IC (volume $V_{\rm IC}$) is given by $n = V_{\rm IC} \cdot N_0/V_{\rm m}$, where $N_0$ is Avogadro's number and $V_{\rm m}$ is the molar volume. Then we can calculate the particle flow, $dn$ in response to the fountain pressure $dP$

$$\frac{dn}{dP} = -V_{\rm IC}\frac{N_0}{V_{\rm m}^2}\frac{dV_{\rm m}}{dP}. \tag{4}$$

From the London equation (Supplementary Equation 1), we can calculate the increase in particle number, $\Delta n$ in the IC in response to a temperature increase $\Delta$T, noting that $\frac{dV_{\rm m}}{dP}$ is negative.

$$\Delta n = -V_{\rm IC}\frac{N_0}{V_{\rm m}^2}\frac{dV_{\rm m}}{dP}\rho S\Delta T. \tag{5}$$

We calculate $dV_{\rm m}/dP$ from the values in Reference [2] and obtain $dV_{\rm m}/dP$= -1.8$\times$10$^{-12}$ m$^3$ $\cdot$ mole$^{-1}\cdot$ Pa$^{-1}$ at 22 bar, and -1.2$\times$10$^{-11}$ m$^3$ $\cdot$ mole$^{-1}\cdot$ Pa$^{-1}$ at 0 bar. At 0.5 $T_{\rm c}$, the inflow of particles into the IC is of order $7 \times 10^{16}$ particles $\cdot$ K$^{-1}\cdot$ $\Delta$T at 22 bar and $5.5 \times 10^{16}$ particles $\cdot$ K$^{-1}\cdot$ $\Delta$T at 0 bar, using values of $V_{\rm m}, S$ from Supplementary Table 2. For a nominal temperature rise of 25 $\mu$K, this would result in the transfer of $\sim$ 10$^{12}$ particles undergoing superflow into the IC to

equalize the chemical potential. If we consider only the free volume of $^3$He above the sinter, $V_{\rm free}$ (approximately $2.8\times10^{-7}$ cm$^{-3}$) then at a temperature of $0.5T_{\rm c}$, where $\rho_{\rm s}/\rho$ ∼0.7, the fraction of superfluid in the free volume above the sinter depleted by the superflow into the IC is estimated to be $\sim 10^{12}/(N_0 \cdot \rho_{\rm s}/\rho \cdot V_{\rm free}/V_{\rm m})$ is small (of order $10^{-9}$) and undetectable. Thus, the signal received at the HEC fork in response to a pulse applied at the IC fork (or the inverse) cannot be due to direct depletion of the superfluid.

We can estimate the rate of superflow $dn/dt$ in response to an applied heat pulse,

$$\frac{dn}{dt} = v_{\rm c} \cdot h \cdot w \frac{N_0}{V_{\rm m}} \frac{\rho_{\rm s}}{\rho} \tag{6}$$

where $h, w$ are the channel height and width. We find that if the flow is limited by the critical velocity $v_{\rm c} \sim 1.5 \times 10^{-2}$ [m/s] achieved across the full channel, then $dn/dt \sim 10^{18}$ particles·s$^{-1}$, so the flow would cease after a time interval of a few $\mu$s. Since pulses are applied with a duration of 100 sec, then it must be true that the average flow velocity of the superflow is less than critical.

We can calculate the average superflow velocity during the pulse $v_{\rm s}$ from Supplementary Equations 5 and 6. We start by rewriting Supplementary Equation 5

$$\frac{dn}{dt} = \frac{dn}{dT} \cdot \frac{dT}{dt} = -V_{\rm IC} \frac{N_0}{V_{\rm m}^2} \frac{dV_{\rm m}}{dP} \rho S \frac{\Delta T}{\Delta t} \tag{7}$$

and equate it to Supplementary Equation 6 but with $v_{\rm s}$ instead of $v_{\rm c}$.

$$\frac{dn}{dt} = -V_{\rm IC}\frac{N_0}{V_{\rm m}^2}\frac{dV_{\rm m}}{dP}\rho S\frac{\Delta T}{\Delta t} = v_{\rm s}\cdot h\cdot w\frac{N_0}{V_{\rm m}}\frac{\rho_{\rm s}}{\rho}. \tag{8}$$

We then solve for the average superflow velocity $v_{\rm s}$ in the channel

$$v_{\rm s} = -\frac{1}{\rho_{\rm s}/\rho}\frac{1}{h\cdot w}\frac{V_{\rm IC}}{V_{\rm m}}\cdot\frac{dV_m}{dP}\rho S\frac{\Delta T}{\Delta t} \tag{9}$$

At 22 bar and $0.5T_{\rm c}$, $v_{\rm s} \sim 3\times10^{-10}$ m/s.

Once the high drive to the excited fork reverts to its ambient value, the fountain pressure in the heated chamber will be at its maximum. This pressure differential across the length of the channel will initiate normal fluid counterflow into the non-heated chamber. In Supplementary Table 1, we list the normal volume flow at 22 bar as $3.8\times10^{-15}$ $\rm m^3$/s, corresponding to a normal flow velocity of $1.15\times10^{-6}$ m/s. This is much larger (by a factor of 1000) than the superflow calculated in Supplementary Equation 9. Since this normal flow must be accompanied by a corresponding counterflow of superfluid, we use the fact that the net particle flux must be zero (aside from the relaxation of the fountain pressure by thermal diffusion) to equate currents. Thus $j_{\rm s} = \rho_{\rm s}\cdot v_{\rm s}$ = -$\rho_{\rm n}\cdot v_{\rm n}$ and we find $v_{\rm s}$ = $0.5\times10^{-7}$ m/s. Near $T_{\rm c}$, since $\rho_{\rm s}$ decreases, the superfluid velocity must increase strongly.

Both far away and close to $T_{\rm c}$, we find that $v_{\rm s}$ as calculated in the channel is dominated by the hydrodynamic counterflow and not the flow to build up the fountain pressure. This brings

forward two important conclusions. First the flow is maximum in the flow channel because this is where temperature differences are largest and the cross-sectional areas are the smallest. Thus, the average flow velocity will be maximised in this region, and consequently it is likely that the anomalous behavior will originate from phenomena in this region. The average flow velocity likely understates the velocities achieved in this geometry. In the vicinity of a sharp corner, the flow velocity will be enhanced. Establishing that it could approach critical values would require numerical calculations and detailed knowledge of the geometry beyond the scope of this paper.

A puzzling observation is that there do not appear to be significant differences in the normal or superfluid velocities when comparing 0 bar and 22 bar calculations. There is of course the substantial difference in mean free paths at these two pressures. It is possible that the estimates of normal flow obtained by analogy from the flow in much larger geometries is inadequate. Indeed, a comparison between experiments and theoretical results in Ref[3] show that the models **underestimate** the reduction in effective viscosity in the long mean free path limit attained at low temperatures and low pressure in that experiment. It is also likely that the normal flow in response to the fountain pressure is reduced by effective surface roughness. The micro machined surfaces of the channel used here are likely to be smoother than the epoxy surfaces in the mass flow experiments, so a direct extrapolation of the effective viscosity at play in these two experiments may be problematic. These observations highlight the need for a comprehensive model for thermal conduction under conditions of strong confinement.

**Supplementary Note 3. Received pulse observed at the HEC Fork.**

In Supplementary Figure 4, we show a sampling of the temperature excursion in the HEC fork $\Delta T^{\mathrm{HEC}*}/T_c$ against the corresponding temperature excursion in the IC fork $\Delta T^{\mathrm{IC}}/T_{\mathrm{c}}$ following a pulse to the IC fork. The seven pulses shown at each pressure were recorded sequentially at the lowest temperatures achieved (where signal to noise is better) while warming at $\sim 25$ $\mu$K hr$^{-1}$ at 22 bar, 0.62 bar and 0 bar. At 22 bar when we applied pulses to the IC fork, we observe that the thermal response on the HEC fork is proportional to the IC fork's temperature excursion. To within the accuracy of our measurement the response is linear for the observed $\sim 1\%$ temperature excursion of the HEC fork from its ambient temperature. At 0 bar and 0.62 bar, we typically applied larger pulses to the IC fork. Upon analysis, the response in the HEC fork is seen to be non-linear. The linearity is restored when the temperature response in the HEC fork is below $\sim 1\%$ of the ambient temperature. As the temperature increases from the lowest temperature measured, the $Q$ of the forks decreases strongly near $T_{\mathrm{c}}$. The lowered $Q$ is accompanied by a diminution of the signal-to noise, especially in the HEC fork. It appears that the current induced in the flow channel has some critical value above which the response ceases to be linear or that the transmission of surface bound excitations in the bulk is subject to some limitation. However, quantification of the temperature dependence of the limit of the linear behavior would require better signal to noise than is available in this experiment.

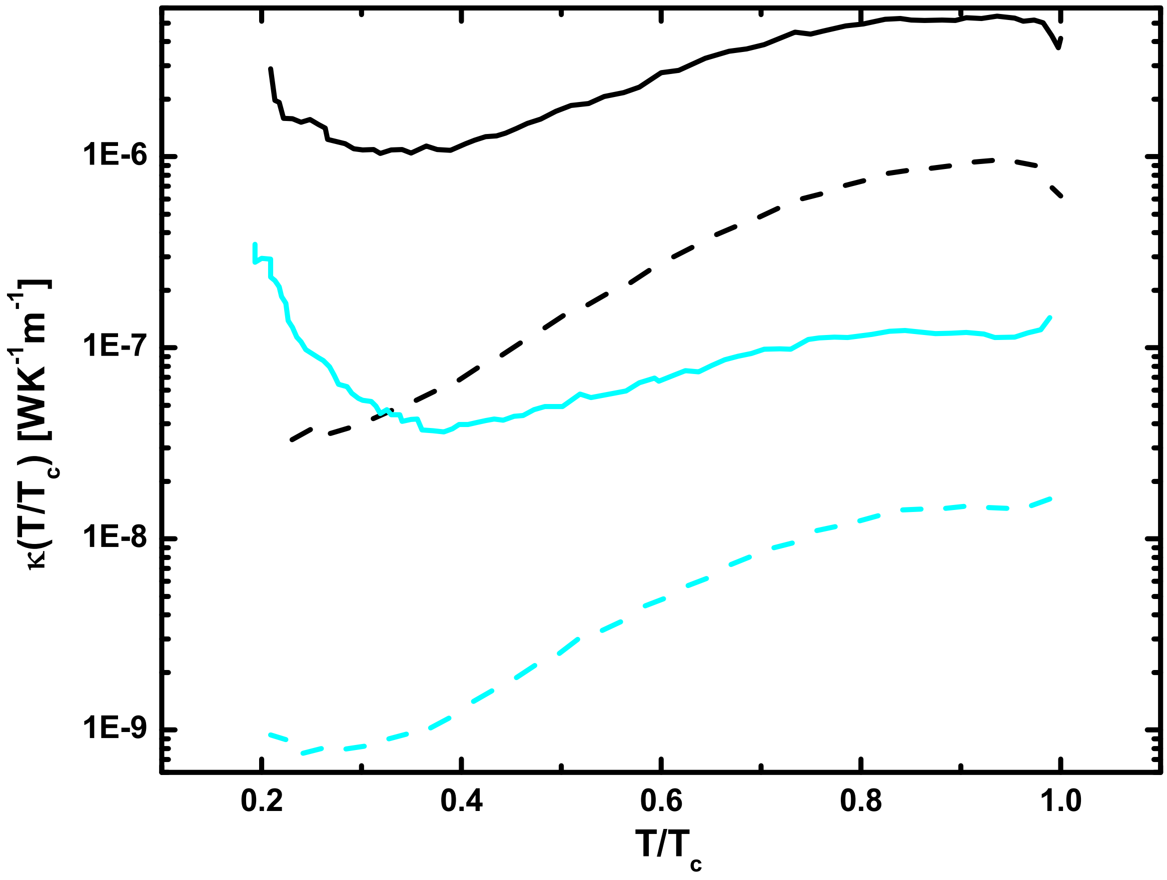


Supplementary Figure 1: **Calculated Hydrodynamic Thermal Conductivity.** The calculated hydrodynamic thermal conductivity at 0 bar (blue traces) and at 22 bar (black traces) due to normal liquid heat flow as described by Supplementary Equation 3. Dashed lines show behaviour calculated using the simplified form for the effective viscosity $\eta_{\mathrm{EFF}}$ and solid lines correspond to the behaviour for the viscosity as shown in Supplementary Reference [3].

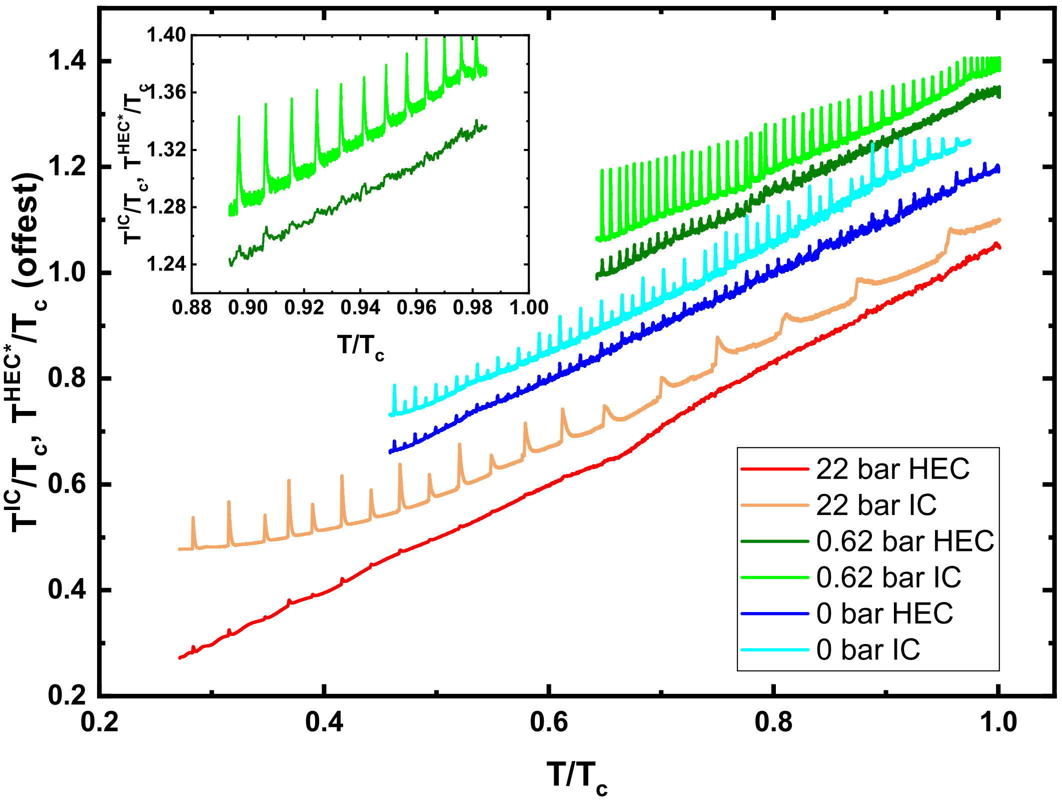


Supplementary Figure 2: **Heat pulses to IC and responses in both forks.** Data on warming showing the response ($T^{\mathrm{HEC}*}$) in the HEC (darker colours) to pulses applied in the IC ($T^{\mathrm{IC}}$ - lighter colours) at three pressures (0.62 bar - green, 0 bar - blue, 22 bar - red). At 0 bar the response (dark blue) to pules applied to the IC fork (cyan) is seen at all temperatures, but is strongest at low temperatures (relative to pulse size). The response at 0.62 bar (dark green) displays a weak minimum around 0.9 $T_{\mathrm{c}}$ (aligned with the location of the minimum in mean free path (see inset). The response is strongly attenuated at 22 bar above 0.6 $T_{\mathrm{c}}$ once the mean free path drops below 6 $\mu$m. $T^{\mathrm{HEC}*}$ is defined by calibrations of the $Q$ against the $T_{\mathrm{MCT}}$. Offsets are applied to the data for clarity.

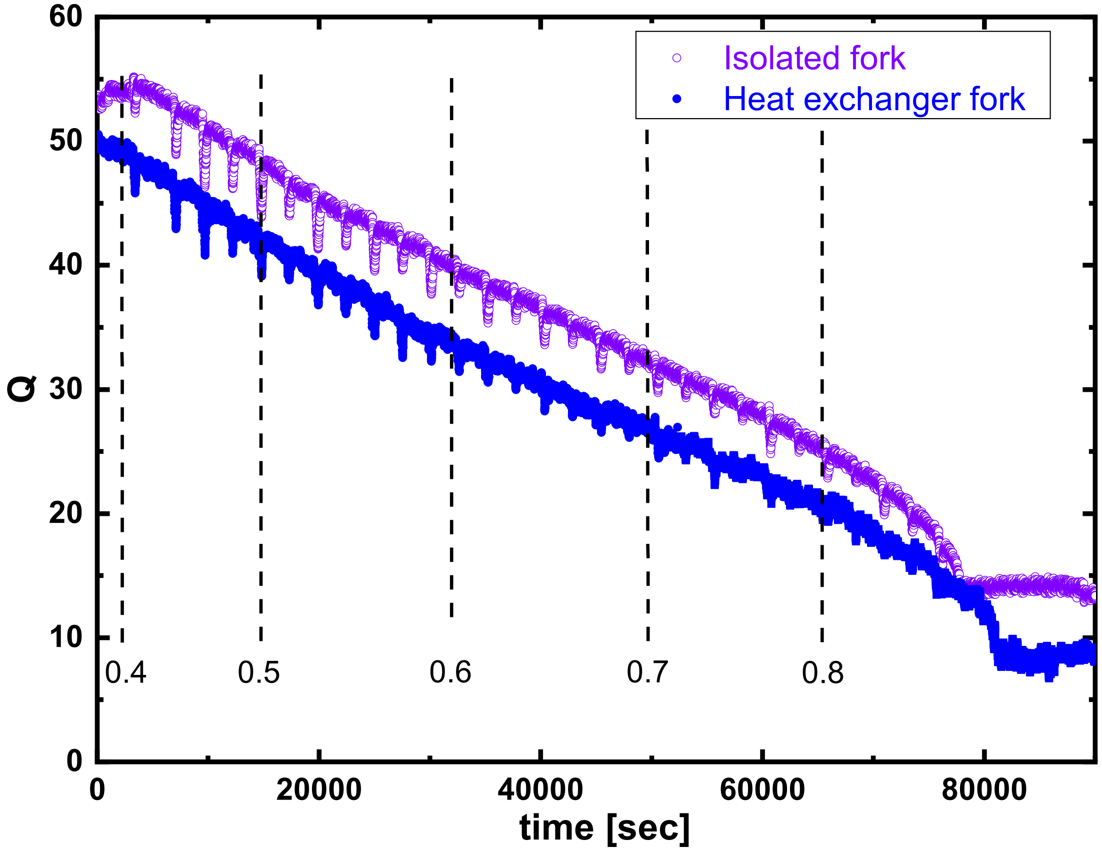


Supplementary Figure 3: **Pulses applied to HEC and responses in both forks.** Pulses applied to the HEC fork (blue) along with the simultaneous response observed in the IC fork (purple) at 0 bar. The figure illustrates that the anomalous heat current is bi-directional. The vertical lines show $T/T_c$ at that time point.

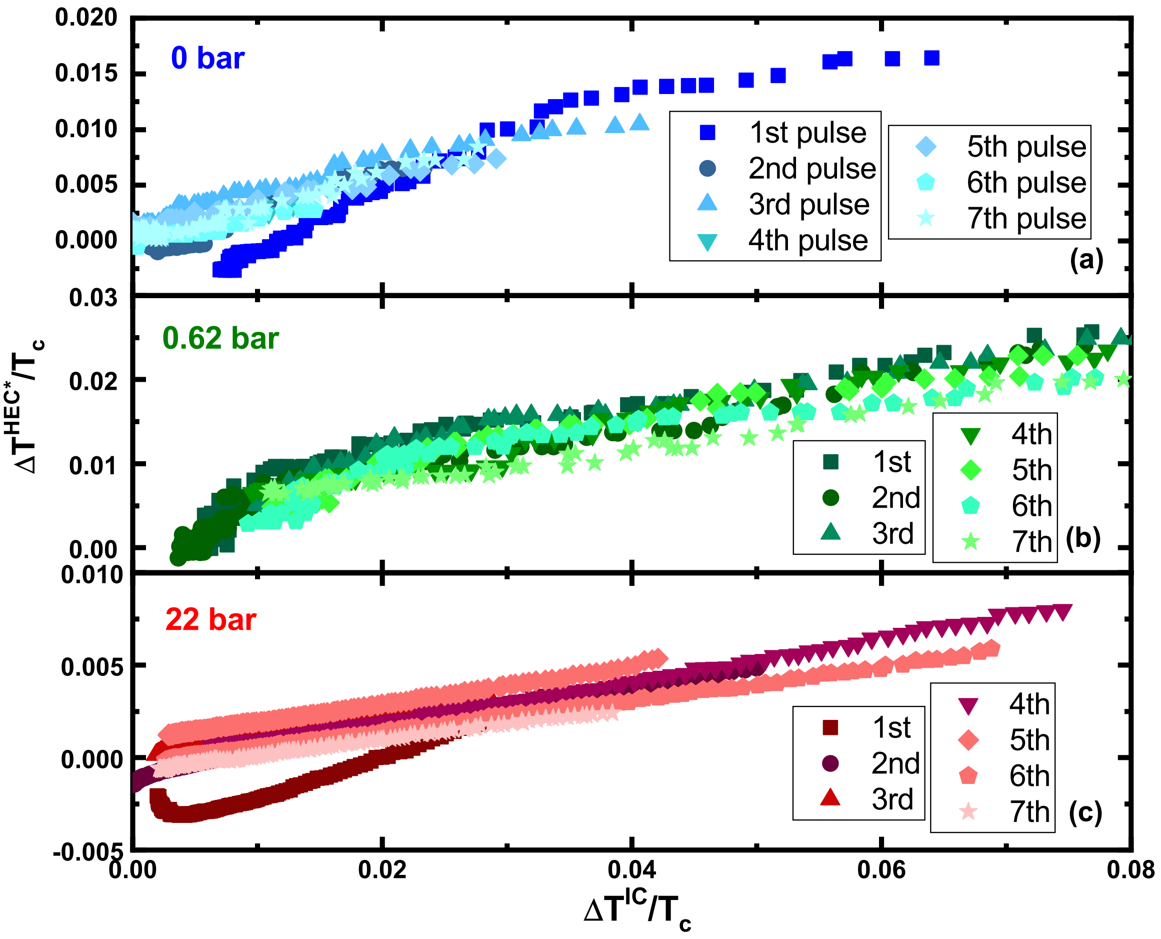


Supplementary Figure 4: **Low temperature limited linear response of HEC following pulses to IC.** The inferred local temperature excursion $\Delta T^{\mathrm{HEC}*}/T_{\mathrm{c}}$ vs $\Delta T^{\mathrm{IC}}/T_{\mathrm{c}}$ following pulses to the IC fork at (a) 0 bar, (b) 0.62 bar and (c) 22 bar. The response is seen to be linear for small $\Delta T^{\mathrm{HEC}*}/T_{\mathrm{c}}$. The linear region is limited to $\approx 1\%$ of $T_{\mathrm{c}}$ excursion. The pulses shown are the first few applied following cooling to the lowest temperature at each pressure. This first heat pulse (squares) was followed by a second (circles) through the seventh pulse (stars).

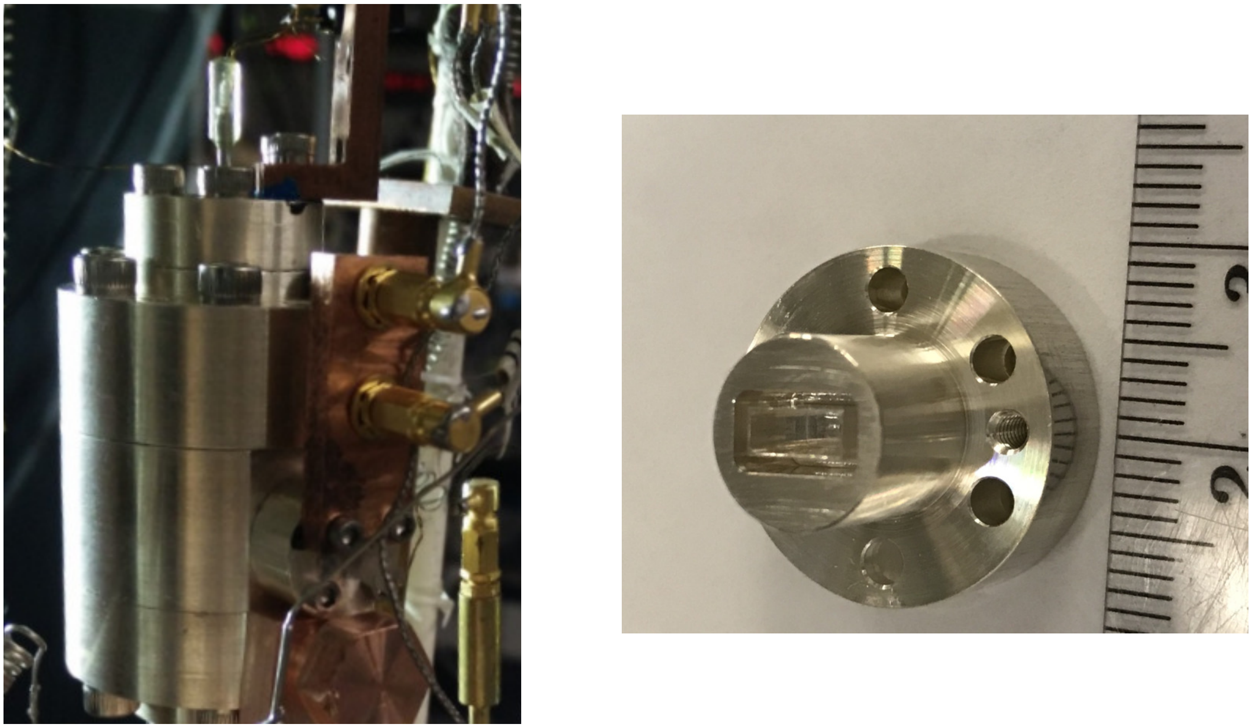

(a) (b)

Supplementary Figure 5: **Details of Experimental Cell.** a) image of the assembled cell structure. b) image of the coin-silver cavity holder prior to gluing in the cavity. Each small division is 1 mm on scale.

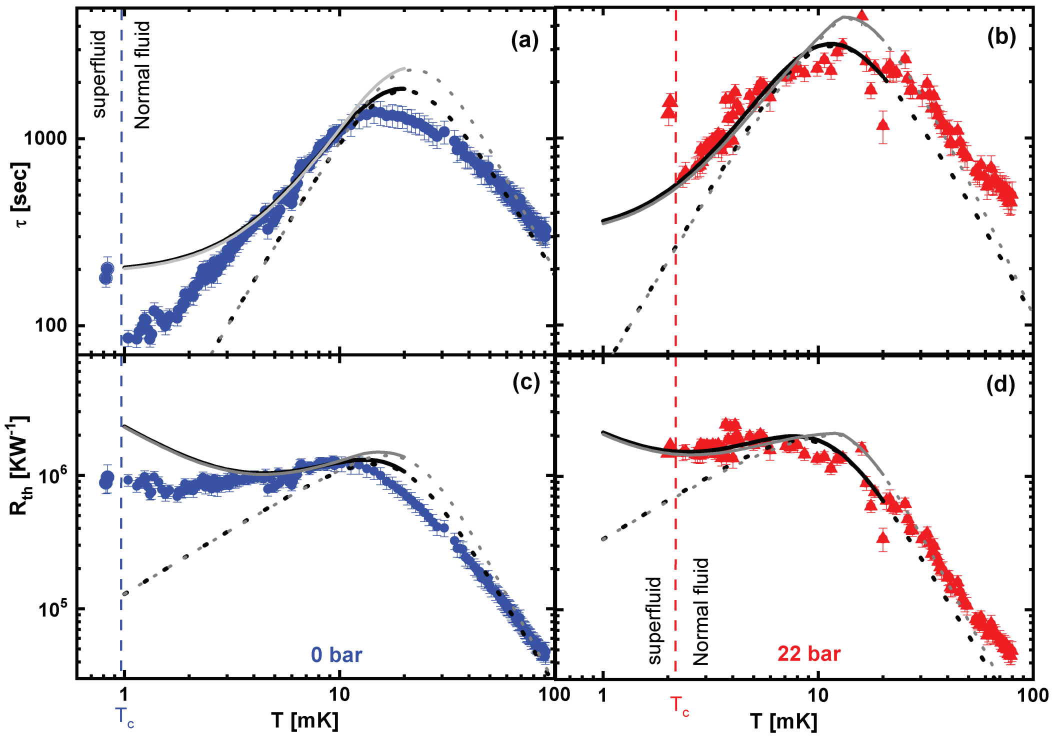


Supplementary Figure 6: **Measured and calculated relaxation times and thermal resistances.** The measured thermal relaxation times (a) and (b) and calculated thermal resistances (c) and (d) at 0 bar (blue circles) and 22 bar (red triangles) in the normal state (compare to main text Figure 3). The calculated bulk behavior: black dotted lines for the bulk fluid thermal resistance in the channel in parallel with Kapitza resistance for sheet[4] and grey dotted lines for the bulk fluid thermal resistance in the channel in parallel with thermal boundary resistance for sinter[5]. Solid lines show the behavior expected for an isotropic distribution of point scatterers in the channel that give rise to a limiting mean free path of 1.1 $\mu$m, in parallel with the Kapitza resistance for sheet[4] (black) and sinter [5] (grey). $T_c$ is marked by vertical dashed lines in blue (0 bar) and red (22 bar)). The bulk calculations reference measured specific heat[2], thermal conductivity[6].

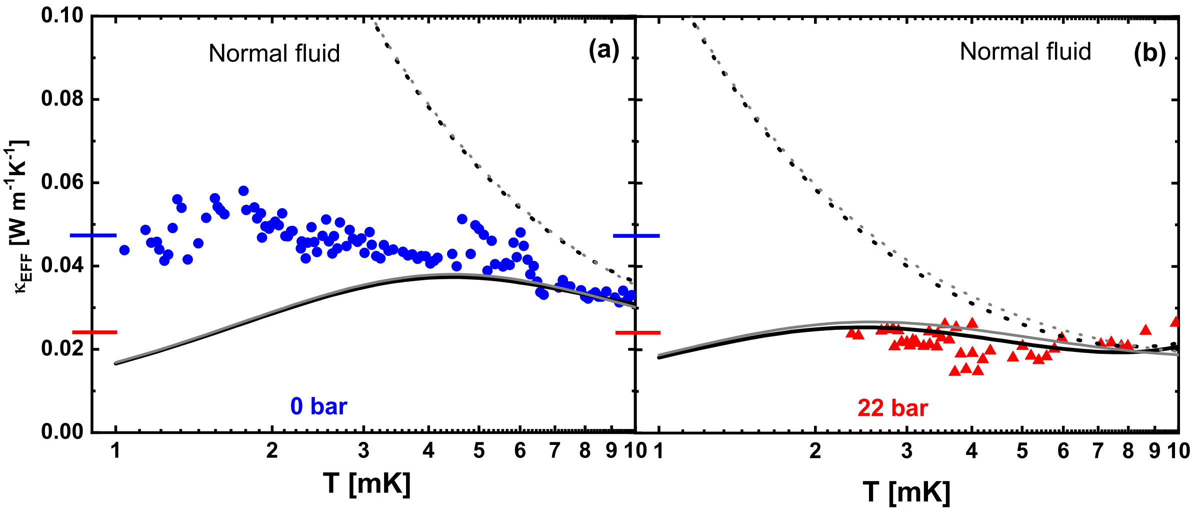


Supplementary Figure 7: **Effective thermal conductivity compared to bulk and impurity-limited conductivities.** The thermal conductivity $\kappa_{\mathrm{EFF}}(T)$ at 0 bar (blue circles) and 22 bar (red triangles) below 10 mK in the normal state calculated from geometrical parameters and $R_{\mathrm{th}}(T)$. Also shown are the calculated bulk behaviors (dotted lines) exhibiting the $T^{-1}$ dependence of inelastic scattering of Bogoliubov quasiparticles. The solid lines show the thermal conductivity expected for a distribution of point scatterers that give rise to a mean free path of 1.1 $\mu$m. The horizontal lines define the values for $\kappa_{\mathrm{EFF}}(T)$ at 0 bar (blue 0.047$\pm$0.005 WK$^{-1}$m$^{-1}$), and 22 bar (red 0.024$\pm$0.003 WK$^{-1}$m$^{-1}$) as $T_{\mathrm{c}}$ is approached in the normal state. Black lines (parallel Kapitza resistance of a sheet[4]) and grey lines (parallel Kapitza resistance of a sinter[5]) show that the choice of Kapitza resistance model does not significantly affect the value of $\kappa_{\mathrm{EFF}}(T)$.

**Supplementary References:**


1. London, F. *Superfluids, Volume II, p87* (John Wiley & Sons, 1954).

2. Greywall, D. 3He specific heat and thermometry at millikelvin temperatures. *Physical Review B* **33**, 7520 – 7538 (1986).

3. Einzel, D. and Parpia, J. M. Finite-Size Effects and Shear Viscosity in Superfluid $^3$He-$B$. *Phys. Rev. Lett.* **58**, 1937–1940 (1987).

4. Cousins, D. J and Fisher, S. N. and Guénault, A. M and Pickett, G. R. and Smith, E. N and Turner, R. P. $T^{-3}$ Temperature Dependence and a Length Scale for the Thermal Boundary Resistance between a Saturated Dilute $^3$He-$^4$He Solution and Sintered Silver, *Phys. Rev. Lett.* **73**, 2583–2586 (1994).

5. Andres, K. and Sprenger, W.O. Kapitza resistance measurements between 3He and silver at very low temperatures. *Proceedings of LT14, Matti Krusius and Matti Vuorio, eds.,* **1**, 123 – 126 (1976).

6. Greywall, D.S. Thermal conductivity of normal liquid 3He. *Physical Review B* **29**, 4933 - 4945 (1984).
